# AVA Spring Meeting, Qinetiq, Farnborough April 15, 2019

**DOI:** 10.1177/2041669519853911

**Published:** 2019-06-10

## The Geoffrey J Burton Lecture: Colour Appearance and Spatio-Chromatic Vision

Sophie Wuerger

University of Liverpool, UK

The response of the human visual system depends on a multitude of image features, such as the wavelength (colour) of the visual stimulus and its spatial frequency content. I will review current models of colour appearance, in particular, unique hues as a tool to measure appearance and its potential applications. Secondly, colour is always associated with a spatial component, and chromatic and spatial information is encoded simultaneously, most likely in the same neurones. I will review experimental evidence on spatio-chromatic processing (discrimination of spatial orientation and blur discriminability) consistent with this idea. Recent experiments on spatio-chromatic contrast sensitivity assessed on HDR displays will be discussed.

## Keynote Talk: Coding Strategies in Binocular Vision and Stereopsis

Paul Hibbard

University of Essex, Colchester, UK

Three-dimensional vision from binocular information relies on the encoding and interpretation of binocular disparities—the differences in position of corresponding points between the left and right retinal images. The first stage of this process appears to enact a binocular cross-correlation, so that binocular disparity is estimated as the shift in position that produces the greatest similarity in samples taken from the two images. These calculations are performed based on an absolute coordinate system, in which disparity is defined relative to positions in the retina. In contrast, the perception of depth depends on relative disparities, in which the disparity of a point is defined relative to that of other points in the image. The perception of depth is thus limited by both the initial encoding of absolute disparities through cross-correlation, and the subsequent encoding of surface structure through relative disparity calculations. These stages can explain the effects of disparity discontinuities, and the presence of disparity masks, on perceived depth. The encoding of surface structure can be performed by a cyclopean energy model, which extends the traditional binocular energy model to take account of the visual system’s dependence on relative disparity, and invariance over absolute disparity.

## Keynote Talk: Visual Perception in the Presence of Nystagmus

Matt Dunn

School of Optometry and Vision Sciences, Cardiff University, UK

Nystagmus is an incurable condition causing constant oscillatory eye movements. If the condition develops in infancy, adults with nystagmus are not directly aware of an oscillating visual scene. The presence of visual stability, despite constant eye motion, provides a uniquely dynamic perceptual scenario, and the opportunity to better understand visual reference frames and motion perception in humans. The condition usually occurs in conjunction with an afferent visual system pathology, but even in isolated cases, visual acuity is usually reduced. Recent advances have demonstrated that visual acuity alone is insufficient to fully explain the subjective perceptual experience of infantile nystagmus (IN). This talk will provide an introduction to the condition and an update on recent attempts to measure visual function in patients with nystagmus.

## Talks^1^

## Five Dimensions of Labelled Line Theory

David Rose

School of Psychology, University of Surrey, Guildford, UK

The notion that sensory pathways are ‘labelled’ has been a centrepiece of neural coding theory and of visual psychophysics for many years. However, like any theory that survives for a long time, it has developed into a complex conceptual structure, to enable it to account for a wide variety of empirical phenomena. Qualitative textual analysis was used to survey and understand its usage[s] within visual psychophysics over recent decades. This revealed at least five conceptual dimensions to the theory-space within which researchers have positioned different variations of the theory: (a) Level of description—labels may be envisaged as operating at, or as bridging, such levels as the neural, informational and mental. (b) Stage or locus along the sensory pathway—labels have been postulated to exist at retinal and at several cortical locations. (c) Plasticity or speed of change—labels have been suggested to change rapidly (e.g., with eye movements), slowly (perceptual learning) or not at all. (d) Phenomenal quality or cognitive or semantic—a label may be proposed to account for a particular subjective experience, for the cognitive category of the stimulus, or for some other aspect of its ‘meaning’ or significance (semantic associations, affordances, etc.). (e) Accuracy and scale of measurement—labels may or may not be measurable on nominal, ordinal, interval or ratio scales, and (hence) may or may not have degrees of imprecision or uncertainty attributable to them. Examples are presented and discussed. It is concluded that, for each use of ‘labels’ to explain a given set of experimental data, their location along these five dimensions should be specified in full detail.

## Validation of a Novel Gaze-Contingent Perimeter With High-Speed Eye Tracking

Nikita Thomas, Jennifer H. Acton, Jonathan T. Erichsen and Matt J. Dunn

School of Optometry and Vision Sciences, Cardiff University, UK

Microperimetry enables gaze-contingent measure of visual field sensitivity in individuals with unstable fixation. Microperimeters use partial gaze-contingency (PGC), where stimuli are presented at the last known eye position at a speed of 25 Hz, which is insufficient for highly unstable fixation (infantile nystagmus). We have developed a continuously gaze-contingent perimeter (CGC; stimulus position updates each frame) using the EyeLink 1000 Plus eye tracker (130 Hz). Here, we compared the test–retest variability of CGC against PGC and no gaze-contingency (NGC) in normally sighted observers and hypothesised that variability is unaffected by gaze-contingency, providing proof-of-concept for the instrument’s use. In 33 participants (three visits), sensitivity was measured (10° grid) under CGC, PGC or NGC conditions. Test–retest intervals were defined as the 5th and 95th percentile range of sensitivities from follow-up visits and they were not significantly different across the conditions (repeated-measures analysis of variance), *F*(2, 67) = 1.86, *p* = .17, η_p_^2 ^= 0.027, 95%CI [0.01, 0.19]. Four cases are also presented that demonstrate the ability of our instrument to detect visual field loss. We hypothesised that CGC better detects the physiological blind spot than PGC and NGC in infantile nystagmus. This was measured as the ‘verticality’ of the slope delineating the decline in sensitivity to represent the edge of the blind spot (1/absolute steepness), which was significantly steeper for CGC compared to PGC and NGC (repeated-measures analysis of variance), *F*(2, 16) = 19.51, *p* < .01, η_p_^2 ^= 0.91, 95%CI [0.51,0.86]; pair-wise comparisons Bonferroni; all *p* < .01. The ability of our instrument to detect sensitivity loss with validated outcomes supports its potential for accurate visual field assessment in all individuals with poor fixation.

## Posters^1^

## The Development of Attentional Control and Reducing Attentional-Capture

Rumandeep Hayre, Lucy Cragg and Harriet Allen

University of Nottingham, UK

**Objectives:** Attentional control reduces attentional-capture by distractions. It is unclear at what age top-down control is used and whether this skill is beneficial or costly to children who are beginning to use it.

**Methods:** One hundred twenty nine participants (5–6-, 9–11- and 18–25-year-olds) reported the tilt (left or right) of a target-line among two other upright lines. Each line was presented in a coloured patch. Trials were preceded by a coloured cue which matched (predictive) or did not match (unpredictive) the coloured patch of the target-line. There were three blocks where trials were: (a) Mostly Predictive, (b) Mostly Unpredictive and (c) Neutral (cues unpredictive throughout). A singleton-distracter was presented on 41.67% of trials in each block to measure attentional-capture.

**Results:** A significant Block × Cue-Guidance (neutral vs. predictive) × Cue-Type (predictive vs. unpredictive) × Age-Group interaction was found, *F*(2, 126) = 7.47, *p* = .001. Cue-use significantly differed from Neutral in 9–11-year-olds in the Mostly Predictive block only and 18-25-year-olds in both blocks. Five- to 6-year-olds were faster on predictive than unpredictive trials in the Mostly Predictive block but this cue-use did not differ from Neutral. All participants resisted attentional-capture better on predictive, relative to unpredictive trials, *F*(1, 126) = 9.43, *p* = 0.003; *t*(128) = −2.55, *p* = .012, two-tailed.

**Conclusions:** Top-down control is still developing in early-childhood and becomes ‘adult-like’ by late-childhood. Nine- to 11- and 18- to 25-year-olds used top-down control similarly in the Mostly Predictive block but differed in the Mostly Unpredictive block. Five- to 6-year-olds sometimes used top-down control in the Mostly Predictive block but found this difficult to sustain. Regardless of age, top-down control is beneficial as all participants used predictive cues to reduce attentional-capture.

## Effects of Simulated Central Scotoma on Binocular Shape Discrimination Hyperacuity in Central and Peripheral Vision

Anna Zolubak and Luis Garcia-Suarez

University of Plymouth, UK

**Background:** In healthy observers, binocular vision is typically superior to monocular vision; however, in certain conditions, for example, in Age-related Macular Degeneration (AMD), it can be worse than monocular vision (Tarita-Nistor, González, Markowitz, & Steinbach, 2006; Valberg & Fosse, 2002). One of the AMD-sensitive functional indicators is Shape Discrimination Hyperacuity (SDH) (Wang, Wilson, Locke, & Edwards, 2002), yet it is unclear how central scotoma affects its binocular performance.

**Methods:** We examined SDH in monocular, binocular and simulated scotoma conditions. Monocular scotoma was simulated in healthy participants (*N* = 4, *M*_age_ = 27 years) using self-designed goggles. In a 2-interval forced choice (2IFC) staircase paradigm, we measured modulation thresholds to differentiate between a sinusoidally deformed circle (radial frequency pattern) and a circle. Stimuli were achromatic and yellow-blue with radii of 1.2°, 7.5° and 15°. The simulated scotoma occluded 1.2° stimuli and 60% of the 7.5° stimuli but did not interfere with 15° patterns.

**Results:** The binocular SDH did not differ from the best monocular SDH in the achromatic, *F*(4, 45) = 1.34, *p* = .269, and the yellow-blue condition, *F*(2, 18) = 2.48, *p* = .57. We observed binocular inhibition at 1.2°(*n* = 3), and at 7.5° and 15°(*n* = 2). Furthermore, with simulated scotoma on the better-seeing eye, we found central binocular facilitation in the achromatic condition at all eccentricities (*n* = 2).

**Conclusions:** For most observers, results indicate no binocular gain in SDH. There was a large interindividual variability in the effects of stimulated scotoma, especially in partial occlusion. AMD patients also experience variable scotomas’ effects on binocular vision; hence, further studies should explore mechanisms underlying inter-individual differences in long-term scotomas’ effects on SDH.

## Note

1 Abstracts reproduced here are those where the presenter requested publication in i-Perception as part of the meeting registration process. Other talks and posters were also presented at the meeting and are archived on the AVA meeting website.
